# Current translational potential and underlying molecular mechanisms of necroptosis

**DOI:** 10.1038/s41419-019-2094-z

**Published:** 2019-11-12

**Authors:** Tamás Molnár, Anett Mázló, Vera Tslaf, Attila Gábor Szöllősi, Gabriella Emri, Gábor Koncz

**Affiliations:** 1 Department of Immunology, Faculty of Medicine, University of Debrecen, Debrecen, Hungary; 2 Doctoral School of Molecular Cellular and Immune Biology, University of Debrecen, Debrecen, Hungary; 3 MTA-DE Cell Biology and Signaling Research Group, Faculty of Medicine, University of Debrecen, Debrecen, 4032 Hungary; 4 Department of Dermatology, Faculty of Medicine, University of Debrecen, Debrecen, Hungary

**Keywords:** Necroptosis, Chronic inflammation, Oncogenesis

## Abstract

Cell death has a fundamental impact on the evolution of degenerative disorders, autoimmune processes, inflammatory diseases, tumor formation and immune surveillance. Over the past couple of decades extensive studies have uncovered novel cell death pathways, which are independent of apoptosis. Among these is necroptosis, a tightly regulated, inflammatory form of cell death. Necroptosis contribute to the pathogenesis of many diseases and in this review, we will focus exclusively on necroptosis in humans. Necroptosis is considered a backup mechanism of apoptosis, but the in vivo appearance of necroptosis indicates that both caspase-mediated and caspase-independent mechanisms control necroptosis. Necroptosis is regulated on multiple levels, from the transcription, to the stability and posttranslational modifications of the necrosome components, to the availability of molecular interaction partners and the localization of receptor-interacting serine/threonine-protein kinase 1 (RIPK1), receptor-interacting serine/threonine-protein kinase 3 (RIPK3) and mixed lineage kinase domain-like protein (MLKL). Accordingly, we classified the role of more than seventy molecules in necroptotic signaling based on consistent in vitro or in vivo evidence to understand the molecular background of necroptosis and to find opportunities where regulating the intensity and the modality of cell death could be exploited in clinical interventions. Necroptosis specific inhibitors are under development, but >20 drugs, already used in the treatment of various diseases, have the potential to regulate necroptosis. By listing necroptosis-modulated human diseases and cataloging the currently available drug-repertoire to modify necroptosis intensity, we hope to kick-start approaches with immediate translational potential. We also indicate where necroptosis regulating capacity should be considered in the current applications of these drugs.

## Facts

- Necroptosis is closely associated with the pathogenesis of many human diseases.
- The in vivo appearance of necroptosis indicates that both caspase-independent and caspase-dependent mechanisms control this cell death pathway.
- More than 70 human molecules play a role in the regulation of necroptosis.
- More than 20 approved drugs have the potential to regulate necroptosis.

## Open Questions

- How can we monitor and regulate necroptosis in human diseases?
- What are the main molecular targets in caspase independent regulatory mechanisms of necroptosis?
- How effective can the off-label use of already approved drugs in necroptosis-driven diseases be?

## Introduction

The development and homeostasis of multicellular organisms depends on the balance between cell proliferation and cell death. In the past few years new regulated cell death pathways have been discovered and classified^1^. One of these tightly controlled inflammatory cell death pathways – necroptosis – has come to the center of attention because of its known contribution to the pathogenesis of many diseases^1,2^.

**Fig. 1:**
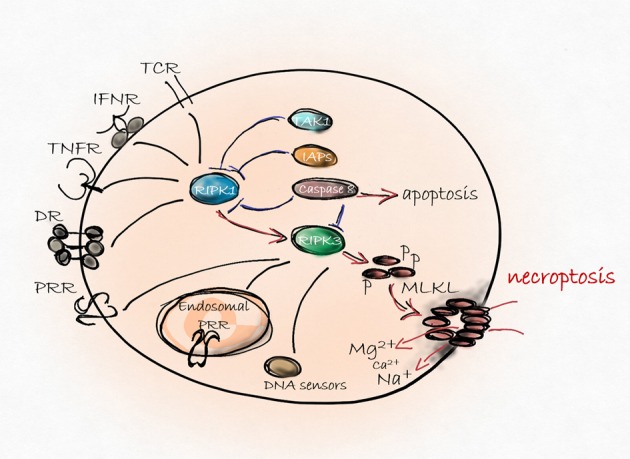
Backbone of necroptosis signaling. Various extra - or intracellular signals activates the RIPK3 protein directly or through RIPK1. RIPK3-mediated phosphorylation induces MLKL membrane translocation and consequently, ion influx results in necroptosis^147^. Survival signals through upregulation of IAPs or activation of TAK1 kinase pathway blocks RIPK1-induced signaling and protects cells from unwanted necroptosis. Caspase-8-mediated cleavage of pro-necroptotic RIPK1 and RIPK3 ensures the dominance of immunologically silent apoptosis to immune stimulant necroptosis

Many death-, pattern recognition-, DNA binding-, adhesion, and dependence-receptors, immune reactions, pathogens and various drugs have been identified as necroptosis triggers^1,3^. Necroptosis utilizes a signaling pathway requiring the involvement of receptor interacting protein kinase 3 (RIPK3)^4^, mixed lineage kinase domain-like protein (MLKL)^5^ and upon stimulation of death receptors (DR)^2^ RIPK1. RIPK3 oligomerization and its subsequent phosphorylation allows the RIPK3-MLKL interaction and the double phosphorylation of MLKL by RIPK3^6^. After this step, MLKL forms oligomers and translocates to the plasma membrane to execute necroptosis (Fig. 1). Generally, necroptosis requires inhibition of caspases^3,7^ or the absence of the pro-caspase-8-activating adaptor Fas-associated protein with death domain (FADD)^8^, demonstrating the crucial role of the apoptotic platform in the negative regulation of necroptosis. Active caspases block necroptosis^2^ preferentially through the cleavage of RIPK1^9^, RIPK3^3,10^, and cylindromatosis (CYLD) protein^11^ which acts as the de-ubiqutinase enzyme of RIPK1. During DR-mediated signaling, inhibitors of apoptosis proteins (IAPs) initiate the ubiquitination of RIPK1 and this process favors cell survival^12^. Blockage of IAPs or the subsequent events of IAP-induced signaling strongly support necroptosis^13^. Various molecular pathways have been documented as regulators of downstream necroptotic events beside MLKL-mediated membrane rupture, but the complexity of the signaling and regulation network of necroptosis are still not fully understood.

The immunological outcome of cell death can be classified as anti-inflammatory or pro-inflammatory and tolerogenic or immunogenic^1^. Dominance of apoptosis ensures the tolerogenic outcome of cell death under physiological conditions. When apoptosis signaling is blocked, necroptotic pathways are activated and the dying cells have the potential to initiate innate immune responses via production of damage associated molecules (DAMPs) resulting in an inflammatory response^14^. Signaling in necroptotic cells also supports the cross priming capacity of dendritic cells (DCs)^15^.

In this review our goal was to understand the molecular background of necroptosis in humans and to find potential points of clinical intervention. We summarized how the expression, posttranslational modification, and localization of necroptotic molecules are regulated and what the interaction partners of the necrosome complex are. Finally, we provide an overview of drugs, which are already used in the clinic and have been shown to affect necroptosis.

## Necroptosis involved in human diseases

Currently, necroptosis is mainly documented in various in vivo mice models^16,17^, but regulated necrosis contributes to the pathogenesis of many human diseases (Table 1). Both up and down-regulation of necroptosis and misregulation of the apoptosis-necroptosis transition which modifies the immunological outcome of cell death contribute to the evolution of degenerative disorders, autoimmune processes, inflammatory diseases or the immune surveillance of tumors.

**Table 1:** Necroptosis related diseases in human

Disease	Molecular changes in possible diagnosis
Lipid storage disorders	
Niemann–Pick disease ^224^ *	Increased expression of RIPK1 and RIPK3 in cerebellar tissue.
Skin disorders	
Toxic epidermal necrolysis ^58^ *	Upregulated RIPK3 expression and elevated MLKL phosphorylation in skin tissue sections
Cutaneous vasculitis^50^	Strong phospho-MLKL signals in infiltrating tissue neutrophils in biopsy specimens
Psoriasis^50^	
Lichen Planus^56^	Detection of highly upregulated RIPK3 and increased phosphorylation of RIPK3 and MLKL
Systemic lupus erythematosus^56^	
Cardiovascular diseases	
Chronic Heart Failure^38^ *	Elevated expression of RIPK1and RIPK3, increased RIPK3 and MLKL phosphorylation, downregulation of active caspase-3 and 7
Coronary artery disease^43^	Patients with CAD plasma RIP3 levels were significantly higher than controls
Unstable atherosclerosis^40^	High RIPK3 and MLKL expression. Increased phosphorylation of MLKL.
Abdominal Aorta Aneurysm^41,42^	Elevated levels of RIPK1 and RIPK3 in AAA tissue
Neurodegenerative disorders	
Multiple Sclerosis^32^	High RIPK1 and RIPK3 expression. Increased phosphorylation of RIPK1 and RIPK3. Reduced expression of active Caspase-8.
Amyotrophic Lateral Sclerosis^35,36^	Elevated levels of RIPK1, RIPK3 and MLKL, increased RIPK1 and p-MLKL phosphorylation in both microglia and oligodendrocytes primarily localized in the white matter.
Alzheimer’s disease^33,34^	Detection of activated RIPK1
Spinal cord injury^37^	After SCI, strong RIP3-, phosphorylated-MLKL- (pMLKL) and HMGB1-immunoreactivities were detected.
Gastrointestinal diseases	
Alcoholic liver disease^17^	Increased expression of RIPK3
Non alcoholic fatty liver disease^44,45^	Increased RIPK3 and MLKL expression
Drug-induced liver injury^46^	Elevated phosphorylation of MLKL
Crohn’s disease^17^	Increased expression of RIPK3
Primary biliary cholangitis^47^	Elevated expression of RIPK3, phosphorylation of MLKL, insoluble aggregates of RIPK1, RIPK3 and MLKL
Ulcerative colitit^49,50^	Strong phospho-MLKL signals in infiltrating tissue neutrophils in biopsy specimens
IBD in children^48^	Increased expression of RIPK3 and MLKL and reduced caspase-8 in patient’s tissue
Autoimmune diseases, Immunodeficiency	
Immunodeficiency, arthritis and intestinal inflammation^62,63^	Loss-of-function mutations in RIPK1 detected with exome sequencing
Renal diseases	
Acute kidney injury^51^	Phosphorylation of RIPK3 and MLKL
Autosomal dominant polycystic kidney disease^53^	Phosphorylation of RIPK3 and MLKL
Kidney ischemia-reperfusion injury^52^	Phosphorylation of MLKL
Autoimmune vasculitis in the kidney^54^	Phosphorylation of MLKL in neutrophils
Skeletal system diseases	
Kashin‐Beck disease^60^	High RIPK3 expression and necrotic cell death morphology in the middle zones of KBD samples. Negative staining for caspase‐3
Dental diseases	
Chronic periodontitis^61^	Elevated levels of RIPK1, phosphorylated RIPK3, MLKL, phosphorylated MLKL and cFLIP_L_ in gingival tissues
Pulmonary diseases	
Chronic obstructive pulmonary disease^59^	Increase in expression of RIPK3 and PINK1 using confocal imaging

Some physiological processes such as alteration of glucose level, oxygen deprivation or immune reactions resulted in elevated RIPK3 expression allowing in vivo emergence of necroptosis. Hyperglycemia (35–40 mM glucose) markedly enhanced the expression of RIPK3 in various cell lines and primed cells for necroptosis^18,19^. Similarly, upregulated expression of RIPK1, RIPK3 and MLKL, and increased RIPK1/3 complex formation have been observed in hypoxic cells^20–22^. At the same time caspase-8 mRNA, functioning as a negative regulator of necroptosis, was reported to be transiently decreased following the deprivation of oxygen and glucose (OGD)^23^. These processes are also involved in brain injury caused by hypoxia-ischemia and OGD-induced necroptosis^24,25^. Type I^26–28^ and type II^27,29^ interferons have been published to induce increased expression of RIPK3, while constitutive IFNβ signaling was demonstrated to increase the intracellular level of MLKL^28^. CD8+T lymphocytes can trigger both apoptosis and necroptosis, which make these cells capable of killing tumor cells, even those that escaped apoptosis^30^. T cell-mediated necroptotic cytolysis also plays a role in activation induced cell death, and can be critical in the development of autoimmune reactions^31^.

### Upregulation of necroptosis in human diseases

Necroptosis takes part in the pathogenesis of human *neurodegenerative disorders*, such as Multiple Sclerosis (MS)^32^, Alzheimer’s disease (AD)^33,34^, and Amyotrophic Lateral Sclerosis (ALS)^35,36^. Defects in the activation of caspase-8 were demonstrated in the pathologic process of MS. Additionally, activated forms of RIPK1, RIPK3 and MLKL were detected in the cortical lesions of human MS samples^32^. Activated RIPK1 as a marker of necroptosis was also observed in human AD brains correlating positively with Braak stage and negatively with brain mass and cognition^33,34^. In ALS samples, multiple biochemical hallmarks of necroptosis including increased levels of RIPK1, RIPK3 and MLKL and elevated pRIPK1 and pMLKL were detected in both microglia and oligodendrocytes. Importantly, pMLKL was primarily localized in the white matter, where demyelination was found^35^. In spinal cord injury strong RIPK3 expression and MLKL phosphorylation were detected^37^.

In certain *cardiovascular diseases, such as* chronic heart failure (HF) cell loss and subsequent deterioration of contractile function is associated with elevated expression of RIPK1, RIPK3, and pRIPK3. On the other hand, the expression of caspase-8 was downregulated suggesting activation of necroptosis signaling. MLKL expression did not differ among the control and HF groups; however, pMLKL were present in all HF samples, which is in contrast to the controls where this was almost undetectable^38^. A genetic variant in the RIP3 promoter region was associated with increased RIPK3 transcription, which contributed to the poor prognosis of HF patients^39^.

In humans with unstable carotid atherosclerosis, expression of RIPK3 and MLKL was increased, while the phosphorylation of MLKL was detected in advanced atheromas^40^. In patients with abdominal aorta aneurysm, the tissue showed elevated levels of RIPK1 and RIPK3 proteins^41,42^. In coronary artery disease higher plasma RIPK3 levels were detected than in controls^43^.

Regarding *gastrointestinal diseases*, increased RIPK3 expression was detected in liver biopsies from patients with alcoholic liver disease^17^, while both RIPK3 and MLKL expression was increased in non-alcoholic fatty liver diseases^44,45^, as well as elevated MLKL phosphorylation in drug-induced liver injury^46^. High levels of RIPK3 and MLKL phosphorylation were also detected in the liver biopsies of patients with primary biliary cholangitis, in contrast with its low hepatic expression in healthy controls^47^. Similarly, increased levels of RIPK3 were documented in the terminal ileum of patients with Crohn’s disease^17^ and elevated RIPK3 and MLKL levels were observed in inflamed tissues of inflammatory bowel disease (IBD) and allergic colitis patients, whereas the expression of caspase-8 in these tissues was reduced^48^. The migration of human neutrophils to sites of inflammation was found to activate the RIPK3-MLKL pathway: a strong pMLKL signal was observed in infiltrating tissue neutrophils in samples collected from patients with cutaneous vasculitis, ulcerative colitis, and psoriasis^49,50^.

Phosphorylation of MLKL molecules was also detected in human acute kidney injury biopsies^51^, in biopsies taken immediately after excision for transplantation^52^ and in autosomal dominant polycystic kidney disease^53^ representing involvement of necroptosis in *renal disorders*. Antineutrophil cytoplasmic antibody (ANCA) induces neutrophil extracellular traps via necroptosis and causes subsequent endothelial cell damage. ANCA-associated vasculitis exhibited a specific p-MLKL staining in glomerular neutrophils in human kidney biopsies^54^.

Concerning *skin diseases*, human biopsy samples obtained from patients with Lichen Planus (LP) and Systemic lupus erythematosus (SLE) confirm the role of necroptosis in their development. RIPK3 and MLKL activation was demonstrated in podocytes in renal biopsies from patients with lupus nephritis^55^. LP and SLE tissue sections showed enhanced epidermal expression of phosphorylated RIPK3^56^. B cells from SLE patients also significantly displayed high expression levels of necroptosis-related genes^57^. As we already mentioned, phosphorylation of MLKL in the infiltrated human neutrophils was also found in cutaneous vasculitis and psoriasis^49,50^. Upregulation of RIPK3, and elevated MLKL phosphorylation were observed in the skin samples from patients with toxic epidermal necrolysis in correlation with unwanted necroptosis and subsequent inflammation^58^.

Expression of RIPK3 and dynamin-related protein 1 (Drp1) was increased in lung tissue homogenates collected from patients suffering from chronic obstructive pulmonary disease, proving the role of necroptotic cell death in *pulmonary diseases*^59^. In Kashin–Beck disease (KBD) necroptosis dominates as a cell death mechanism in the middle zone of cartilage from KBD children^60^. Necroptotic cell death is involved in the progression of chronic periodontitis, as gingival tissue in patients showed increased levels of RIPK1, RIPK3, and MLKL, as well as increased phosphorylation of MLKL^61^.

Although RIPK1 is one of the key molecules required for execution of necroptosis, patients with its complete deficiency due to homozygous mutations suffered from recurrent infections, early-onset of IBD and progressive polyarthritis. In vitro, cells with RIPK1 deficiency showed impaired mitogen-activated protein kinase activation and cytokine secretion and were prone to necroptosis^62,63^.

### Role of necroptosis in cancers

An increasing number of studies have been published about the importance of necroptotic cell death in anti-cancer therapies, which have been extensively reviewed in recent papers^64,65^.

Briefly, both pro- and anti-tumoral effects have been demonstrated following necroptosis in cancer development and progression. The anti-tumoral effect of necroptosis has been shown in many types of cancer in which the expression of RIPK3^66,67^ or MLKL^68^ was silenced or polymorphisms in their coding genes lead to modified expression of necrosomal components^66,69^. In general, necroptosis resistance of cancer cells is a common process, and escape from necroptosis was suggested to be a potential hallmark of cancer, similar to the escape from apoptosis^64^. Additionally, effective anti-cancer agents trigger immunogenic cell death, inducing the killing of the transformed cells and provoking the members of innate and adaptive immune system to attack. Beside the massive release of DAMPs, necroptotic cells create a great possibility to trigger the activation of CD8 + T cells via cross presentation^15,70^. The dual ability of necroptosis to activate innate and adaptive immunity simultaneously makes this cell death pathway a promising therapeutic target.

However, the tumor-promoting outcome of necroptosis has also been shown. RIPK3 and MLKL expression seems to vary among tissue samples from different subtypes and stages of cancer, and downregulation of necroptosis mediators has also been published in various cancers^71–73^. Upregulated RIPK3 expression is a general phenomenon in tumor necrotic areas playing a critical role in tumor growth and metastasis^74^. Necroptosis-induced inflammation contributes to tumorigenesis and necroptosis can also lead to an immunosuppressive tumor microenvironment^75^. The immune-suppressing environment was associated with necroptosis-induced expression of the chemokine attractant CXCL1^71^. It has also been shown that tumor cells induce necroptosis of endothelial cells, which promotes tumor cell extravasation and metastasis^76^. Thus, we can conclude that necroptosis occurs in different phases during tumorigenesis and plays an ambivalent role in tumor formation.

## Molecular mechanisms in the regulation of necroptosis

To understand the molecular background of necroptosis and to find potential points of clinical intervention we summarize below how the expression, the posttranslational modification, and the localization of key necroptotic molecules (RIPK1, RIPK3 and MLKL) are regulated, while also highlighting the interaction partners of the necrosome complex.

### Regulation the expression level of necroptotic proteins

RIPK3-RIPK3 homodimerization is sufficient to induce necroptosis; after which, its kinase domain stimulates the activation of RIPK3 through cis-autophosphorylation; a prerequisite step for the recruitment of MLKL^77–79^. Thus, RIPK3 dimerization is probably the most critical point of necroptosis induction. Several lines of evidence support the idea that increased expression of RIPK3 can induce its oligomerization and can initiate necroptosis^42,80^. RIPK1 dimerization, and accordingly upregulation of RIPK1, facilitates RIPK3 oligomerization, mainly upon death receptor stimuli.

**Fig. 2:**
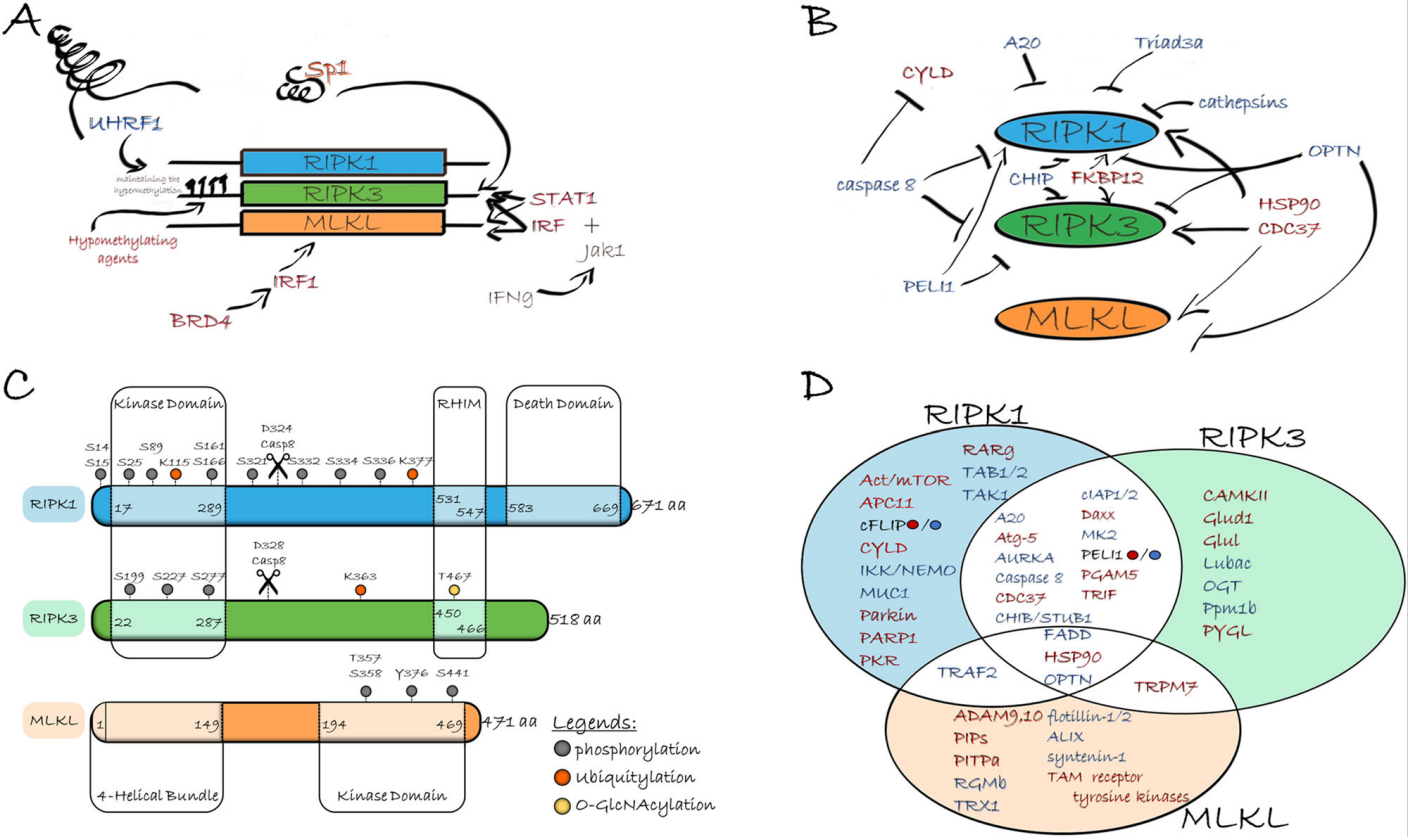
Direct interacting partners of main necroptotic signaling molecules. Sp1 transcription factor increases RIPK3 expression. INFγ-mediated up-regulation of RIPK3 and MKLK level depend on JAK1 kinase, and STAT1 and IRF transcription factors. BRD4 cooperating with IRF1 also increase MLKL transcription. Hypermethylation of the RIPK3 promoter by UHRF1 results in silenced RIPK3 expression. The stability of all RIPK1, RIPK3 and MLKL proteins are increased by HSP90 and CDC37 co-chaperone complex and by FKBP12. The level of both RIPK1 and RIPK3 are down-regulated by caspase-8-mediated cleavage. Cathepsins are also capable of processing RIPK1. A20, CHIP, Optn, PELI1 and Triad3a ubiquitin-ligases mediate K48-linked polyubiquitylation and the subsequent proteasome dependent degradation of: RIPK1, RIPK3 and/or MLKL Upon necroptosis human RIPK1 is autophosphorylated at ser14, ser15, ser161, ser166 and RIPK3 at ser199 and ser227 and ser277. The transient phosphorylation of RIPK1 at ser321 is phosphorylated transiently by TAK1 leads to RIPK1-independent apoptosis and the sustained phosphorylation of RIPK1 by TAK1 at ser321, ser332, ser334 and ser336 induces RIPK1 kinase activation^106^. IKKα/IKKβ also phosphorylate RIPK1 at ser25 and thereby block RIPK1 activity^108,214,215^. Mitogen-activated protein kinase-activated protein kinase 2 (MK2) mediates phosphorylation of RIPK1 at ser321 and ser336 and restrains integration of RIPK1 into the cytosolic death complex^107,216,217^. The phosphorylation at ser89 by a currently unknown kinase inhibits the RIPK1 kinase activity^218^. Ubiquitylation of RIPK1 at Lys115 by PELI^219^ or Lys377 by cIAP1, cIAP2 and Parkin^220^ promotes necroptosis. LUBAC complex and the deubiquitinase CYLD regulates M1 ubiquitination of RIPK1^221^. Lys363 ubiquitylation of RIPK3 leads to its proteasomal degradation. RIPK3 is responsible for the phosphorylation of MLKL at thr357 and ser358. TAM (Tyro3, Axl, and Mer) family of receptor tyrosine kinases phosphorylate MLKL on Tyr376 to facilitate MLKL oligomerization^145^. MLKL is also phosphorylated on Ser441 by a still unidentified kinase^222^. Caspase-8 mediates the cleavage and inactivation of RIPK1 at asp324 and RIPK3 at asp328. O-GlcNAcylation of the RIPK3 at thr467 by OGT prevents necroptosis^223^. Red names indicate interaction partners of RIPK1, RIPK3, MLKL which activate necroptosis, blue marks necroptosis inhibitors

All aspects of necroptotic protein expression are intensely regulated, including their transcriptional activity, the stability of the expressed molecules and their degradation. Specificity protein 1 (Sp1), a zinc-finger transcription factor, directly regulates RIPK3 expression in cancer cells. Knockdown of endogenous Sp1 significantly decreases the transcription of RIPK3, while re-expression of Sp1 restores necroptotic response in vitro^81^. Induction of necroptosis by interferon gamma (IFN-γ) resulted in elevated levels of RIPK3^27^ and MLKL^28,29,82^. This effect was found to depend on janus kinase 1 (JAK1) and its substrates: the signal transducer and activator of transcription 1 (STAT1) and interferon regulatory factor (IRF) transcription factors, pinpointing interferon-stimulated gene factor 3 (ISGF3) as a critical promoter^83^. Bromodomain-containing protein 4 (BRD4), a member of the bromodomain and extraterminal domain (BET) family, has been shown to interact IRF1 and to upregulate MLKL transcription^84^. Oncogenes such as BRAF and AXL have also been implicated in the regulation of RIPK3 expression^67^. The activity of RIPK3 promoter is tightly controlled by methylation^67,85–87^ (Fig. 2a). Ubiquitin-like PHD and RING finger domain-containing protein 1 (UHRF1) is essential for the maintenance of the hypermethylation of the RIPK3 promoter and thus contributes to the silencing of RIPK3 expression in quiescent cells.

Following transcriptional regulation multiple processes control the protein level of necrosome components. The heat shock protein 90 (HSP90) and CDC37 co-chaperone complex increases the stability of all RIPK1^88^, RIPK3^89^, and MLKL^90^ proteins. Consequently, inhibitors of HSP90 facilitated the degradation of these necroptotic components and potently blocked necroptosis^91^. Protein levels of RIPK1 and RIPK3 also decreased in FK506-binding protein 12 (FKBP12) knockdown cells^92^.

On the contrary, cells treated with Hsp70 inhibitors underwent cell death, because Hsp70 enhances the stability of necroptosis antagonists, the RIPK1 regulators: cIAP1/2, x-linked inhibitor of apoptosis protein (XIAP), and the *cellular* FLICE-like inhibitor protein (cFLIP)^93^.

The expression of necroptotic molecules are downregulated by cleavage and proteosomal degradation. The most well-known inhibitor of necroptosis, caspase-8 cleaves both RIPK1^9^, RIPK3^94^, and the necroptosis promoting deubiquitinase CYLD proteins^11^. In macrophages, cathepsins were also reported to be capable of processing RIPK1, which resulted in significant decrease in necroptotic cell death^95^.

Several ubiquitin-ligases mediate K48-linked polyubiquitylation and the subsequent proteasome dependent degradation of necroptotic molecules: RIPK1 is regulated by A20^96^, carboxyl terminus of Hsp70-interacting protein (CHIP; also known as STUB1)^97^, optineurin (Optn)^35^, Triad3a^98^, RIPK3 by CHIP^97^, Optn^35^, E3 ubiquitin ligase Pellino 1 (PELI1)^99^, and MLKL by Optn (Table 2)^35^. Knock down of any of these K48 ubiquitin-ligases increased the sensitivity of necroptosis in both in vitro and in vivo studies. (Fig. 2b).

**Table 2:** Molecules in necroptotic signaling

Interaction partners	Outcome of silencing	Confirmed in KO mice	Interactions with…			Regulatory mechanism
			RIPK1	RIPK3	MLKL	
A20	↑^225,226^	The embryonic lethality of A20 ^−/−^ mice is inhibited by RIPK3 KO^225,227^. A20 protects T cells from necroptosis^225^	+^225^	+^225^		A20 KO elevates RIPK3 K5 ubiquitination and RIPK1-RIPK3 complexes formation^225^, but A20 replaces K63 polyubiquitin from RIPK1 with K48 polyubiquitin, leading to RIPK1 degradation^49^.
ABIN-1	↑^67^	The embryonic lethality of Abin-1^−/−^ mice is blocked by inhibition of RIPK1 or absence of RIPK3^67^.				ABIN-1 is an ubiquitin-binding protein associated with TNFR and A20. Regulates the RIPK1 ubiquitylation/deubiquitylation mediated by LUBAC and pA20^67^.
ADAM9 ADAM10	↓^150^				+ ^150^	MLKL binds with multiple ADAMs to mediate the shedding of cell-surface proteins.
ALIX and syntenin-1	↑^149^				+ ^149^	Phosphorylated MLKL was removed from membranes through ALIX–syntenin-1–mediated exocytosis^149^.
APC11	↓^228^		+ ^228^			APC11 promotes necroptosis induced by TNF/5z-7/Zvad, but not upon TCZ. Interaction with RIPK1 was detected upon RIPK1- dependent apoptosis
Akt ½ mTOR	↓ ^124,125^		+ ^124^			Akt/mTOR activation occurs downstream of RIPK1–RIPK3, it does not affect RIPK1–RIPK3 complex assembly^124,126^
Atg5	↓^128^		+ ^199^	+ ^199^		Atg5 needs to the formation of necrosome membrane that aggregate RIPK1 and RIPK3^128^.
AURKA	↑ ^112^	AURKA inhibitor stimulated MLKL phosphorylation and inhibited the growth of implanted tumors. AURKA and GSK3β are Associated With Poor Prognosis in Human Pancreatic Cancer^112^.	+ ^112^	+ ^112^	-^112^	KO of AURKA enhanced RIPK1-RIPK3 and RIPK3-MLKL interactions. Its kinase activity is required for its anti-necroptotic effect. GSK3β acts as a downstream target of AURKA in necroptosis.
Bax/Bak	↓^229,230^					TNFα and zVAD treatment elevated MLKL in the mitochondrial fraction^229^. CypD-mediated regulated necrosis can be responsible for Bax/Bak-regulated necrosis.
BRD4	↓^84^					BRD4 contribute to the transcription complex to regulate the expression of MLKL^84^.
CAMKII	↓^127^	KO of CaMKII abrogated I/R-induced necrosis and blocked doxorubicin-induced contractile dysfunction, myocardial necrosis and mortality^127^		+ ^127,231^		RIPK3-mediates activation of CaMKII, including direct phosphorylation and indirect ROS-mediated oxidation^127^.
Caspase-2	↑^232^					Caspase-2 KO enhanced the phosphorylation of RIPK1 and MLKL^232^.
Caspase-8	↑^2,233^	Casp8 KO leads to embryonic lethality, but Casp8 KO mice fully viable when bred on RIPK3 KO^7,234^. or MLKL KO^235^.	+ ^4,115^	+ ^4,115^		Caspase-8 cleaves RIPK1^236^, RIPK3^94^ and CYLD to block necroptosis^11^.
c-Cbl	↓^228^		+ ^228^			c-Cbl promotes necroptosis induced by TNF/5z-7/Zvad, but upon TCZ. Interaction with RIPK1 was detected upon RIPK1- dependent apoptosis
CDC37	↓^91^		+ ^91^	+ ^91^		RIPK3 activation requires the activity of an HSP90 and CDC37 cochaperone complex^91^
CHIP/ STUB1	↑^97^	CHIP KO mice showed postnatal lethality with intestinal defects, which is rescued by crossing with RIPK3 KO mice^97^.	+ ^97^	+ ^97^		RIPK3 and RIPK1 expression level is negatively regulated by CHIP E3 ligase mediated ubiquitylation^97^.
CypD	↓^10,127,176,196,237,238^	In vivo analysis in mice suggested the distinctness of CypD-mediated MPT from RIPK1/RIPK3-mediated necroptosis^237^.				.Probably, cyclophilin-D (CypD) and RIPK3 mediate two independent form of programmed necrosis^10,176,227^
CYLD	↓^11,239–242^	Inhibition of CYLD catalytic activity in epidermal keratinocytes could delay the development of inflammatory skin lesions in FADD^E-KO^ mice^241^.				CYLD deubiquitylates RIPK1 (both M1- and K63), facilitating the association of RIPK1 and RIPK3^11,239,243^. CYLD promotes the dissociation of TRAF2 from MLKL^121^.
Daxx	↓^244^		+ ^244^	+ ^244^		RIPK3 phosphorylated Daxx at Ser-668 triggering the nuclear export of Daxx^244^.
Drp1	↓^131,132^ debated in^132,135^					.PGAM5S activates Drp1 by dephosphorylation, Drp1 facilitates mitochondrial fragmentation^131^. but in cell type specific manner^132,135^
ESCRT-III components ESCRT-I components	↑^52,245^					.ESCRT-III machinery (CHMP2A, CHMP4B, VPS4B, IST1) controls the duration of plasma membrane integrity, when MLKL activation is limited or reversed^52,245^
FADD	↑^233^	.Fadd KO mice are fully viable when bred RIPK3 KO^246,247^ or Mlkl KO backgrounds^235,248,249^	+^4,101,240,250^	+ ^4,101,216^	+^251^	FADD functions together with caspase-8 in the repression of necroptotic signaling.
FKBP12	↓^92^	FKBP12 is essential for TNFα-induced systemic inflammatory response syndrome.				Protein levels of RIPK1 and RIPK3 decreased significantly in FKBP12 knockdown cells
cFLIP	↑^7,226^ ↓ ^252^	cFLIP KO (as well as caspase-8 KO or FADD KO) results in embryonic lethality, FLIP KO, FADD KO, RIPK3 KO mice are viable^7,247^	+ ^253^			c-FLIP_L_: procaspase-8 heterodimers inhibit RIPK1 and RIPK3^247,254^. cFLIP_S_ and cFLIP_R_ simply block procaspase-8 activation^252^.
Flottilin1-2	↑^149^	Flotillin-null mice were highly senstitive to TZ-induced SIRS^149^			+ ^149^	Phosphorylated MLKL was removed from membranes through flotillin-mediated endocytosis^149^
Gγ10	↓^157^					In complex with Gβ2 and Src regulates intracellular trafficking of necrosomes^157^
GSK3b	↑^112^	AURKA and GSK3β are associated with poor prognosis in human pancreatic cancer^112^.				Phosphorylation of GSK3β at Ser9 by AURKA suppresses the formation of the RIPK3-MLKL complex.
GLUD1	↓^77^			+ ^77^		Targets of RIPK3, contributing to TNF-induced ROS. GLUL and GLUD1 play a role in using glutamine as a supplementary substrate for the TCA cycle.
GLUL	↓^77^			+ ^77^		
HACE1	0	Increased susceptibility of hace-1 Ko mice to DSS-induced colitis depends on RIPK3^255^				HACE1 is required for RIPK1-dependent apoptosis via TRAF2 ubiquitination. HACE1 KO leads to necroptosis dominance to apoptosis^255^.
HSP70	↑^93^					Hsp70 is sustaining the stability of necroptosis inhibitors, cIAP1/2, XIAP, and cFLIP_S/L_^93^.
HSP90	↓^90,91,256^	HSP 90 inhibitor delayed death in TNF-α–induced SIRS in rats, but not in mice^91^	+ ^91^	+ ^90,91^	+ ^90^	Hsp90 regulates the stability of RIPK1, RIPK3 and MLKL^88,90,174^. and blocks the membrane translocation of MLKL^256^.
HtrA2/Omi	↓^257^	Inhibitor of HtrA2, significantly alleviated DSS-induced colitis^258^	+ ^258^			HtrA2 promoted RIPK1 degradation during necroptosis^258^ and induced monoubiquitination of its substrate UCH-L1 during TNF-induced necroptosis^257^
cIAP1cIAP2	↑^240,259^	.RIPK1 + /− allowed XIAP and cIAP1 double KO to survive past birth, and prolonged cIAP2 and cIAP1 double KO survival^13,260^	+ ^240,261^	+ ^261^		.cIAP1 and cIAP2 mediates RIPK1 ubiquitination, allowing the recruitment of LUBAC^262–264^
XIAP	↑^264,265^	RIPK1 + /− allowed XIAP and cIAP1 double KO to survive past birth^13^ XIAP controls RIPK3-dependent cell death and IL-1β secretion in response to TNF^264^				Loss of XIAP results in aberrantly elevated ubiquitylation of RIPK1 outside of TNFR complex^264^.
IKKα IKKβ	↑^108^	The lethality induced by TNF + TPCA-1 results from both RIPK1 kinase-dependent apoptosis and necroptosis^108^. RIPK3 is activated in Ikkα/β‐deficient livers, but does not control cholestasis^214^				IKKα and IKKβ in addition to their known function in NF-κB activation-directly phosphorylate RIPK1^108,214^
IKK/NEMO	↑^266,267^	.IEC-specific FADD KO combined with RIPK3 KO prevented colitis development in NEMO IEC-KO mice^268,269^	+ ^266^			NEMO inhibits necroptosis by binding to ubiquitinated RIPK1^267^, blocks the RIPK1-caspase-8 interaction, activates NF-kB^266^.
IPMK IPTK IPPK	↓^142,143^					Phosphorylated inositol products dissociate the auto-inhibitory region from MLKL. IP kinases needs to MLKL oligomerization and membrane localization^142^.
IFNAR1	↓^83^	IFNAR1-deficiency protects against LPS/zVad induced septic shock^83^.				IFNAR1-deficient macrophages displayed greatly reduced IRF9 transcript levels^83^.
IRF1	↓^270^					IRF1 contributes to IFNγ-dependent and also IFNγ-independent necroptosis^270^.
IRF9	↓^83^					IRF9 KO macrophages were highly resistant to necroptosis^83^.
JAK1 Stat1	↓^27,271^					RIPK1-RIPK3 complex requires JAK1/ STAT dependent transcription^27^.
LRRK2	↓^228^		+ ^228^			LRRK2 promotes necroptosis induced by TNF/5z-7/Zvad, but upon TCZ. Interaction with RIPK1 was detected upon RIPK1- dependent apoptosis^228^.
Lubac complex (HOIP, HOIL1, sharpin)	↑^104,226,265^	Absence of HOIP HOIL or Sharpin results in RIPK1-kinase activity-dependent apoptosis and necroptosis in various tissues. Co-deletion of caspase-8 with RIPK3 or MLKL prevents these phenotypes as well as RIPK1 kinase-dead knockin^104,260,272–275^	+ ^265^	+ ^221^		HOIP and HOIL1 mediate ubiquitination of RIPK1^265^. The generated linear ubiquitin-chain and LUBAC recruits TAK1 complexes and NEMO to the receptor complex^243,276^
MKRN1	↑^277^					MKRN1 depletion facilitates necrosome formation independently of FADD^277^.
MK2	↑^107,216^	MK2 inactivation greatly sensitizes mice to TNF-induced lethal shock^216^.	+ ^216,217^	+ ^217^		Phosphorylation of RIPK1 on S321 or Ser336 by MK2 limits RIPK1 activation^216^, RIPK1 autophosphorylation and the RIPK1-FADD-caspase-8 interaction^107,217^
MUC1	↑^114^		+ ^114^			MUC1 interacts with RIPK1 and inhibits necroptosis by modulating the phosphorylation of RIPK1 at Ser166^114^.
OGT	↑^223^	CLP induced lethal sepsis in the *absence of Ogt in macrophages, RIPK3* deficiency rescued it^223^.		+ ^223^		RIPK3 *O*-GlcNAcylation on T467 downregulates necroptosis, blocks RHIM-mediated protein interaction through steric hinderance^223^
OPTN	↑^35^	Optn KO oligodendrocytes were sensitized to TNFα-induced necroptosis. Optn double KO with RIPK1^D138N/D138N^ or with RIPK3 were resistant^35^.	+ ^35^			RIPK1 K48 ubiquitination and degradation was slower in Optn KO MEFs. Expression levels of RIPK1, RIPK3 and MLKL, were all increased in Optn KO mice^35^.
Otulin	↑^278^	Otulin^C129A/C129A^ mice cause embryonic lethality, it was prevented by triple KO of caspase-8 and RIPK3^278^.				The main role of OTULIN is to maintain LUBAC function by suppressing its auto-ubiquitination^278^.
Parkin	↓^279^		+ ^220^			Parkin is an E3 ubiquitin ligase involved the K63 ubiquitination of RIPK1 to promote the activation of NF-κB and MAPKs^220^, but parkin knockdown protected cells from zVAD-induced necroptosis^279^.
Parp1	↓^130^ debated in^280,281^		+ ^130^			.Parp1 is an effector downstream of RIPK1/RIPK3^130,281^. Debated in: Parp1 activation is rather a consequence of necroptosis^128,129^
PDC	↓^134^			+ ^134^		RIPK3 activates PDC by phosphorylating PDC-E3. The activation of PDC increases aerobic respiration, which generates ROS^134^.
PELI1	↓↑^99^	In toxic epidermal necrolysis the expression level of PELI1 decreases^99^.	+ ^99,219^	+ ^99^		.PELI1 ubiquitinates RIPK1 (K115) promoting necroptosis, but K363 ubiquitylation of RIPK3 leads to its degradation in proteasome^99,219^
PGAM5	↓^131^ debated in^132,135^		+ ^131^	+ ^131^		.Upon necrosis induction, PGAM5S activates Drp1 by dephosphorylation (S637) causing mitochondrial fragmentation^131^., but it is cell type specific^132,135^
PIPs	↓^138^					.PIPs as critical binders of MLKL are required for plasma membrane targeting and permeabilization in necroptosis^138,139^
PITPα	↓^144^				+ ^144^	PITPα facilitates MLKL oligomerization and plasma membrane translocation.
PKR	↓^27,83^		+ ^27^			.IFNs transcriptionally activate PKR, which then interacts with and phosphorylates RIPK1 to initiate necroptosis^27,83^
PPM1b	↑^113^	*Ppm1b* protects mice from TNF-induced SIRS through dephosphorylating RIPK3^113^.		+ ^113^		Ppm1b prevents RIPK3 autophosphorylation in resting cells^113^.
PYGL	↓^77^			+ ^77^		Target of RIPK3, contributing to TNF-induced ROS. PYGL regulates pyruvate production.
RARγ	↓^115^	RARγ KO mice are protected from TNF + Z-vad induced death^115^.	+ ^115^			RARγ facilitates RIPK1 dissociation from TNF receptor and the formation of death signaling complexes^115^
RelA	↑^282^	Embryonic lethality of RelA KO mice is partially prevented by the KO of RIPK3 or MLKL, and it is fully rescued by the combined ablation of Fadd and RIPK3 or MLKL or RIPK1^K459A^^282^.				RelA KO leads to TNF-induced activation of FADD-dependent apoptosis and RIPK3-dependent necroptosis.
RGMb	↑^122^	Renal tubule-specific RGMB knockout mice exhibited severe tubular injury, after renal ischemia/reperfusion^122^				RGMb inhibits MLKL membrane translocation or membrane binding^122^.
RIPK1	↓↑^78,118^	.Caspase-8/RIPK1 double-knockout animals die shortly after birth, ablation of RIPK3 to triple knockouts, rescues the viability of these animals. Deficiency in either RIPK3 or MLKL prevented the development of skin lesions in RIPK1E-KO mice^117–120^		+ ^4^	+ ^283^	In a kinase-independent function of RIPK1 the RHIM domains of RIPK1 competes with RHIM domain of TRIF or DAI to RHIM-mediated RIPK3 aggregation, but RIPK1 oligomerization is initiative of death domain driven necroptosis^78^.
Sp1	↓^81^					Sp1 specifically binds to RIPK3 promoter and regulates transcription^81^.
SPATA2	↓^284,285^	In contrary to the in vitro data Spata2 deficiency sensitizes mice to SIRS induced by TNFα^221^.				.SPATA2 binds CYLD into the TNF-RSC and to HOIP. SPATA2 KO reduces phosphorylation of RIPK1 and MLKL in TNF‐α‐induced necroptosis^284,285^
Src	↓^157^					Interacting with Gγ10-Gβ2 complex regulates intracellular trafficking of necrosomes^157^
STAT1	↓^27,83,271^	IFN-γ failed to induce Mlkl transcription in Stat1^–/–^ mice^29^				.RIPK1, RIPK3 and MLKL requires JAK1/STAT1-dependent transcription^27,235^
TAB1/2	↑^286^		+ ^259^			.TAB1/2 function to maintain TAK1 activity, which is required for the survival of naive macrophages^286,287^
TAK1	↑^102,103^	Various tissue injuries have been published in the absence of Tak1, These symptoms are associated primarily with apoptosis and were not rescued by *RIPK3* deletion^288^.	+ ^102,103,259^			TAK1 inhibition triggered the degradation of cIAP2, FLIP, and NFκB-p65. TAK1 blocks RIPK1-RIPK3-FADD complex formation^102,111^. Intermediate domain of RIPK1 is phosphorylated transiently by TAK1^106,289^. Downstream targets of TAK1 phosphorylates RIPK1 (see, MK2, IKK, RelA)
TAM kinases	↓^145^	Tyro3,Axl,Mertk tripla KO mice were completely resistant to the TZ-induced SIRS^145^.			+ ^145^	TAM (Tyro3, Axl, and Mer) receptor tyrosine kinases phosphorylate MLKL to protmote MLKL oligomerizatin and necroptosis^145^
TRAF2	↑^121,290^	TRAF2 deletion causes morbidity, RIPK3 KO delays TRAF2 KO mortality^121,291^ and suppressing TRAF2 augments ischemic brain damage through necroptosis mechanism^292^			+ ^121^	TRAF2-MLKL association suppresses the interaction of MLKL with RIPK3^121^.
Triad3a	↑^98^					Triad3a induces K48 ubiquitination and the degradation of RIPK1, FADD and Caspase-8^98^.
TRIF	↓^83,116^	Mice without functional TRIF did not show macrophage loss and elevation of inflammatory cytokines upon LPS/zVad^293^.	+ ^294^	+ ^116,294^		Activates necroptosis through RHIM dependent association of TRIF with RIPK3 kinase^116^.
TRPM7	↓^146^			+ ^146^	+ ^146^	TRPM7 is a target of MLKL for the induction of Ca (2 + ) influx^146^.
TRX1	↑^123^				+ ^123^	TRX1 blocks *necroptosis* by maintaining MLKL in a reduced inactive state^123^.
UCH-L1	↓^128,257^					HtrA2/Omi induces monoubiquitination of UCH-L1^257^
UHRF1	↑^81^					UHRF1 silences RIPK3 expression via promoter hypermethylation. Sp1 initiates RIPK3 transcription in the absence of UHRF1^81^.

### Posttranslational modifications in the regulation of necroptosis

Accumulating evidence suggests that cell death pathways are finely tuned by posttranslational modifications, such as ubiquitination and phosphorylation. Multiple excellent recent reviews go into extensive detail about the role of these processes in necroptosis^100^, therefore we only provide a brief overview of these processes below. These pathways are mentioned in the tables and figures of this manuscript in the interest of providing a comprehensive visual guide to these processes as well (Fig. 2c).

The necrosome is formed due to the phosphorylation driven assembly of RIPK1, RIPK3, and MLKL^4,80,101^. However several phosphorylation steps have been published to inhibit necroptosis, chief among them the transforming growth factor beta-activated kinase 1 (TAK1) complex, which is the most important hub for these necroptosis-dampening signals^102,103^. Various protein complexes are assembled along TNFR signaling; namely the survival (complex I), the apoptotic (complex IIa and IIb) and the necroptosis inducer (complex IIc) complexes. Upon activation TNFR recruits TRADD, RIPK1, TRAF2, TRAF5 proteins. The gathered E3 ubiquitin ligases, cIAP-1 and cIAP-2 molecules, and the linear ubiquitin chain assembly complex LUBAC (consisting of HOIP, HOIL-1L and Sharpin)^104^ polyubiquitinates RIPK1, and modified RIPK1 can now act as a scaffold for TAK1 and the IKK complex^105^ which molecules in many ways block RIPK1-mediated cell death pathways, and thus the formation of complex II:^106–108^ These mechanism are: (1) By inducing the activation of NFκB and MAPK signaling pathways and thereby increasing the transcription of several survival molecules such as cIAP1/2^109^ and FLIP^110^ (2) by blocking the binding of cell death related molecules to RIPK1^111^ and (3) by phosphorylating RIPK1^106,108^.

### Interaction partners of necrosome components

The activity of necrosome components are also mediated by molecular interactions (Fig. 2d). Three molecules, aurora kinase A (AURKA), PPM1b, and HSP90 have been recently identified as binding partners of RIPK3^90,91,112,113^ and/ or RIPK1^91,112^ in resting cells. AURKA^112^ and PPM1b^113^ act as local inhibitors against spontaneous necroptosis, since their silencing induces necroptosis. PPM1b as a phosphatase prevents RIPK3 autophosphorylation in resting cells^113^. AURKA together with its downstream target, Glycogen synthase kinase 3β (GSK3β) regulates the formation of RIPK1-RIPK3 and RIPK3-MLKL complexes^112^. Silencing or blocking of AURKA, or inhibitors of GSK3β result in necroptosis without any other stimuli. Phosphorylation of GSK3β at Ser9 suppresses necroptosis through interfering with the formation of RIPK3-MLKL complex, however the direct targets of GSK3β still have not been identified. The third molecule which associates with RIPK3 in resting cells, HSP90, is required for proper activation of necroptosis. Formation of the HSP90–CDC37 complex is necessary for RIPK1–RIPK3 interaction, thus it mediates RIPK3 activation during necroptosis. Unsurprisingly HSP90 inhibitors can block TNF-induced systemic inflammatory response syndrome (SIRS) in rats^91^. Additionally, membrane tethered mucins have been shown to interact with RIPK1 to block necroptosis in human bronchial epithelial cells in vitro^114^.

The nuclear retinoic acid receptor gamma (RARγ) is released from the nucleus to initiate the formation of cell death signaling complexes by mediating RIPK1 dissociation from TNFR when cIAP activity is blocked. In vitro silencing of RARγ inhibited necroptosis and in vivo results also confirmed that RARγ was essential for TNF-induced RIPK1-initiated apoptosis and necroptosis (Table 2)^115^.

Although RIPK1 initiates RIPK3 activation during death receptor driven necroptosis, it plays an ambivalent role in the regulation of RIPK3 aggregation. Under special circumstances instead of activation, RIPK1 acts to suppress the spontaneous activation of RIPK3 by TIR-domain-containing adapter-inducing interferon-β (TRIF)^116^ or DNA-dependent activator of IFN-regulatory factors (DAI; also known as ZBP1)^78,117^. RIPK3 oligomerization is able to seed a RHIM dependent oligomer and this process is both sufficient and a necessary step in necroptosis. RHIM domains of RIPK1 intrinsically inhibit RHIM-mediated RIPK3 aggregation by competing with the RHIM domain of TRIF or DAI; conversely death domain-driven RIPK1 oligomerization results in RIPK3 aggregation and necroptosis. In vivo results also reveal a kinase-independent function for RIPK1 in inhibiting necroptosis. Caspase-8/RIPK1 double-knockout animals die shortly after birth, however, additional ablation of RIPK3 to make caspase-8/RIPK1/RIPK3 triple knockouts rescues the viability of these animals^117–120^. These data undoubtedly prove the anti-necroptotic activity of RIPK1 under special conditions^78^.

MLKL association with RIPK3 is also suppressed by a constitutive interaction of MLKL with a competitive inhibitor, TRAF2, in resting cells. TRAF2 deubiquitination by CYLD promotes the dissociation of TRAF2 from MLKL and allows necroptosis^121^. Two other molecules inhibit cell death by blocking MLKL association with pro-necroptotic components: Repulsive guidance molecule b (RGMb) inhibits MLKL membrane translocation or membrane binding^122^ and Redox regulator thioredoxin-1 (TRX1) blocks MLKL disulfide bond formation, and through it the critical polymerization of MLKL^123^.

Various molecules have been published to act as downstream targets of RIPK3 and others to regulate MLKL localization and/or activation. RIPK3 constitutes an important upstream kinase of death associated protein (Daxx), triggering its nuclear export. The Akt/mTOR pathway^124–126^, and Ca^2+^/calmodulin-dependent protein kinase II (CaMKII)^127^ are also active effectors of downstream necroptotic signaling. Accordingly, several models suggest that effects on these signaling routes modify necroptotic intensity. Poly [ADP-ribose] polymerase 1 (PARP-1)^128^ (debated in ref. ^129,130^) and phosphoglycerate mutase family member 5 (PGAM5)^131^ (debated in ref. ^132^) have been documented as cell type specific regulators of downstream necroptotic events (Table 2).

### Glucose metabolism and ROS production in necroptosis

Reactive oxygen species (ROS) have long been considered to contribute to necroptosis^49,133–135^. Oxidation of specific cysteine residues in RIPK1 by ROS activates RIPK1 autophosphorylation. A positive feedback loop is generated because silencing of RIPK1 or RIPK3 reduces ROS production. RIPK1 autophosphorylation is also promoted by mitochondrial ROS and is essential for RIPK3 recruitment into the necrosome. However, necroptosis could occur without ROS induction in some cell lines^135,136^.

Metabolic enzymes − human liver glycogen phosphorylase (PYGL), glutamate-ammonia ligase (GLUL), glutamate dehydrogenase 1 (GLUD1) − increase pyruvate production from glycogen or play a role in glutamine catabolism. These enzymes are activated by RIPK3, resulting in enhancement of aerobic respiration and thus likely contribute to TNF-induced ROS production^80^. Pyruvate dehydrogenase complex (PDC) converts pyruvate to acetyl-CoA, and triggers the entrance of metabolic flux into the tricarboxylic acid cycle. Activated RIPK3 in the necrosome enhances PDC activity by phosphorylating the PDC E3 at T135 and plays a major role in increasing aerobic respiration. Based on in vitro studies, activation of these enzymes has additive effects to aerobic respiration and ROS production (Table 2)^80,134^.

### Intracellular localization of necrosome components

The intracellular localization of necrosome components seems to be crucial in the regulation of necroptosis. The RHIM domain of RIPK1 and RIPK3 mediates the assembly of heterodimeric filamentous structures, and the amyloid-like aggregation of RIPK1/RIPK3 complexes^79^. Compromised cluster formation correlated with decreased programmed necrosis. MLKL has also been reported to form SDS-resistant, disulfide bond-dependent polymers during necroptosis and it has been shown that these MLKL polymers were independent of RIPK1/RIPK3 fibers^137^.

MLKL translocation to the cell membrane is an obligatory step in necroptotic signaling. Phosphatidyl-inositol phosphates (PIPs) as critical binders of MLKL are required for plasma membrane targeting of MLKL and subsequent membrane permeabilization in necroptosis^138,139^. Highly phosphorylated inositol products, but not weakly phosphorylated precursors are able to displace the MLKL auto-inhibitory brace region, which is a necessary event for late plasma membrane breakdown and cell death^140,141^. Accordingly, necroptosis requires inositol polyphosphate-specific kinase activity and in cells containing mutant IP kinases, MLKL failed to oligomerize and localize to membranes despite proper RIPK3-dependent phosphorylation^142^. Deletion of inositol polyphosphate multikinase (IPMK), inositol-tetrakisphosphate 1-kinase (ITPK1)^142^ or inositol pentakisphosphate 2-kinase (IPPK)^143^ inhibited necroptosis. Connected to this, phosphatidylinositol transfer protein alpha (PITPα) interacts with MLKL which facilitates MLKL oligomerization and plasma membrane translocation^144^. Following membrane localization TAM (Tyro3, Axl, and Mer) family of receptor tyrosine kinases phosphorylate MLKL to protmote MLKL oligomerizatin and necroptosis^145^. Beside their direct pore forming ability, membrane-localized MLKL regulates transient receptor potential cation channel, subfamily M, member 7 (TRPM7), a non-voltage-sensitive ion channel, for the mediation of Ca^2+^ influx^146,147^.

Once MLKL is membrane associated, all the endosomal sorting complexes required for transport III machinery (ESCRT-III), flotillin-mediated endocytosis and ALIX-syntenin-1-mediated exocytosis act to sustain survival of the cell. The ESCRT-III-driven plasma membrane repair machinery limits the duration of the loss of plasma membrane integrity upon MLKL activation^52,148^, while endo- and exocytosis removes phospho-MLKL from the plasma mebrane^149^. MLKL also forms a complex with multiple membrane metalloproteinases upon necroptotic stimulus. A disintegrin and metalloproteinase (ADAM)-enzymes are activated to mediate the shedding of cell-surface proteins in response to necroptotic stimuli and through this process also play a key role in promoting necroptosis, but only in adherent cells (Table 2)^150^.

RIPK1^151^, RIPK3^152,153^, and MLKL^154,155^ have all been reported to localize to the nucleus and these translocations preceded necroptotic death^154^. RIPK3 and MLKL have been shown to became activated in the nucleus, and after their cooperative nuclear export, they contribute to cytosolic necrosome formation^155^. Following the interaction of RIPK3 and MLKL, the translocation of this complex to mitochondria-associated membranes has also been demonstrated and this relocation was found to be essential for necroptosis signaling^156^. The intracellular trafficking of necrosomes is regulated by the TNF-induced guanine nucleotide-binding protein γ 10 (Gγ10) – Src signaling pathway^157^, however, RIPK1/RIPK3 kinase activity has no direct interaction with Gγ10 or on Src kinase.

## Drugs to regulate necroptosis intensity

In vitro studies prefer to use caspase inhibitors to activate necroptosis, however we still do not fully understand how necroptosis is activated under physiological conditions. The in vivo appearance of necroptosis indicates that in addition to caspase-mediated processes various caspase independent regulatory mechanisms control necroptosis. Drugs affecting either the expression or the activity of necroptosis mediators, or that modify the indirect regulators of necroptosis may have therapeutic potential (Tables 3 and 4).

**Table 3:** Available drugs to modify necroptosis intensity

Drug/Agent	Effect on necroptosis	Mechanism	Cells tested in necroptosis	Application/clinical trial in general
Anthracycline mitoxantrone^195^	↑	Induces MLKL phosphorylation	Inhibits TC1 and EL4 cell lines-induced tumor growth in vivo in mice	Used in chemotherapy in for various cancer
Bortezomib/PS-341^207^	↓	Disrupts the formation of RIPK1-RIPK3 complex through stabilizing of cIAPs	In vitro studies on primary bone marrow- derived macrophages	Bortezomib (PS-341) is used in Multiple Myeloma treatment
Carfilzomib^190^	↓	Inhibits induction of pRIPK3 and pMLKL.	HT-29 cells	Approved on Multiple Myeloma
Cisplatin^196,197^	↑	Induces necrosome formation	In vitro in *various* cell lines^144,196^ and in vivo in rats^197^.	Used in the treatment of numerous human cancers^295,296^
Cyclosporine A^176^	↓	Reduction in necroptosis markers RIPK1 and RIPK3	In vivo rats cerebral ischemia-reperfusion injury.	Widely used immunosuppressive drug
Dabrafenib^185,186^	↓	RIPK3 inhibition by competing with ATP binding	In vitro in normal human hepatocytes and in vivo in mouse models of ischemic injury.	Approved in BRAF-mutant melanoma
Dasatinib^297^	↑	Plays a role in HMGB1-induced necroptosis.	CCC-HEH-2 human embryonic cardiac tissue derived cell lines	Used as an anticancer drug in CML patients
Dexmedetomidine^211^	↓	Inhibition of HMGB1 expression	H9C2 embryonic rat heart-derived cells	Used in the intensive care setting for light to moderate sedation
Diacerein^177^	↓	Decreased renal expression of RIPK3 and MLKL	Prevents necroptosis in acute kidney injury in rats	Registered in some European Union and Asian countries to treat joint diseases
Dimethyl Fumarate^202,204^	↑	Depletion of GSH, increases MAPK and ROS activation, inhibits the Trx1/NFκB axis	Gastrointestinal CT26 and lymphoid cancer cell lines Se-Ax, HH and CEM cells	Used in relapsing-remitting Multiple Sclerosis
Fluorouracil^198^	↑	Reduces cIAP1 protein level, stabilizes binding between RIP1 and RIP3	In vivo xenograft experiments with HT29 cells blocked tumor growth	Used in chemotherapy in for various cancer
Hypomethylating agents (decitabine, 5-azacytidine and RG108)^87^	↑	Restores RIPK3 in cancer cells where RIPK3 had previously been silenced.	Human breast tumor and AML samples	Decitabine and Azacytidine are used in Myelodysplastic syndrome and AML
Interferons, Type I-II^27,29,83^	↑	Increases expression of RIPK3 and/or MLKL	In vivo mice studies in septic model	Used in differnt diseases
lithium^126^	↑	Induces AKT- and mTOR-mediated necroptosis	in vitro RT4 cells and human primary schwannoma cells	Lithium is used as the first line treatment in bipolar disorders
Melatonin^212^	↓	Represses the RIPK3-PGAM5-CypD-mPTP pathway	In vivo mice studies in cardiac ischemia-reperfusion	Used for Jet Lag sleep disorder
Miconazole	↑	Upregulates RIPK3 and MLKL	MDA-MB-231 cells	Anti-fungal medication
oxaliplatin^195^	↑	Induces ATP release in RIPK3 and MLKL expressing cells	Inhibits TC1 and EL4 cell lines-induced tumor growth in vivo in mice	Used in clorectal cancer
Phenhydan ^191^	↓	Suppresses phosphorylation and activation of RIPK1, RIPK3 and MLKL.	in vitro MEFs, L929, NIH3T3, HT-29, U937, and Jurkat mouse and human cell lines	Used as an anti-convulsive drug
Phenytoin^51^	↓	Partial inhibition of RIPK1	HT29 cells and RAW 264 cells, human colon cancer cell lines	Used as anti-arrhythmic class Ib and as anticonvulsant
Pazopanib^189^	↓	Inhibits RIPK1	FADD-deficient Jurkat cells	Approved for renal cell carcinoma and soft tissue sarcoma
Ponatinib^189^	↓	Inhibits both RIPK1 and RIPK3	FADD-deficient Jurkat cells	Approved in some chronic myeloid leukemia and some acute lymphoblastic leukemia
Rapamycin^178^	↓	Inhibits RIP-1 expression	Experimental retinal detachment in rats	Approved for Prevention of transplant rejection in Lymphangioleiomyomatosis, and to prevent restenosis in coronary arteries following balloon angioplasty
SAHA/Vorinostat^208^	↓	HDAC inhibitor, activates NFkB and p38 MAPK; inactivates JNK and Akt kinase; enhances cFLIPL expression	In vitro L929 cells and human neuroblastoma SH-SY5Y cells	Approved for the treatment of Cutaneous T cell lymphoma
Sorafenib^187,188^	↓	Reduces interaction of RIPK1 with RIPK3, inhibits kinase activity of RIPK1 and RIPK3	In vitro various cells and in vivo protects against TNF-induced SIRS and renal ischemia-reperfusion injury	Approved for advanced thyroid and renal cell cancer, hepatocellular carcinoma
Valproic acid^158^	↑	Histone deacetylase inhibitor, induces JNK1 activation and RIPK1 expression	In vitro *rat* PC12 cells	Used in epilepsy and mood disorders^298^

**Table 4:** Components of traditional medicine as necroptosis regulators

Drug/Agent	Effect on necroptosis	Mechanism	Cells tested in necroptosis	Application/ clinical trial in general
Aucubin^193^	↓	Inhibits MLKL and RIPK1 activation	lithium-pilocarpine induced epilepsy rat model in vivo	Component of Eucommia ulmoides Oliv., a traditional Chinese medicine
Bufalin^163^	↑	Increases the expression of RIPK1 and RIPK3	MCF-7 and MDA-MB-231 human breast cancer cells and in a mouse xenograft model of human breast cancer	Bufalin is a component of Chinese medicine. Completed phase II of a clinical trial on pancreatic cancer.
Bulnesia sarmientoi^171^	↑	Induction of RIPK1	Human lung carcinoma cell lines A-549, and H661, normal human lung fibroblast MRC-5	Analgesic, wound-healing and anti-inflammatory medicinal plant
Curcumol^170^	↑	Upregulates the expression of RIPK1 and RIPK3	Human HSC-LX2 cells	Extracted from the roots of the herb *Rhizoma Curcumae*
Emodin^165^	↑	Emodin upregulated the levels of TNF-α, RIP1, RIPK3 and MLKL	Inhibits U-251 glioblastoma cell line proliferation	Compound extracted from traditional Chinese medicines
Genipin^167^	↓	Attenuation of increased levels of RIPK3, RIPK1/RIPK3 complexes and p-MLKL	in vivo acute liver failure model in mice	Major active compound of the gardenia fruit
Gomisin J^299^	↑	Mechanism is not described	Human breast cancer cell lines (MCF7 and MDA-MB-231)	A component of Schisandra chinensis fruit a Chinese herbal medicine
Lycorine^168^	↑	Upregulates RIPK1 and RIPK3 expression	Multiple myeloma cell line ARH-77	Chinese medicinal herb
Matrine alkaloid^166^	↑	Increases RIPK3 expression; increases ROS production	In vitro in CCA QBC939 and Mz-ChA-1 cell lines	Component of the traditional Chinese medical herb Sophora flavescens Ait.
Neoalbaconol^201,206^	↑	Increase of RIPK1/RIPK3 colocalization, down-regulates cIAP1/2 and TNFα receptor-associated factors TRAFs	Nasopharyngeal carcinoma cell line C666-1	Compound isolated from the fungus, Albatrellus confluens
Patchouli alcohol^179^	↓	Down-regulates RIPK3 and MLKL proteins.	DSS (dextran sulfate sodium)-induced mice colitis in vivo	*Pogostemon* (patchouli) leaves used in traditional medicine
Resibufogenin^162^	↑	Upregulation of RIPK3 and phosphorylation of MLKL	In vitro MEF cells, Human CRC cell lines (SW480, HCT-116) and SW480 cells xenografted to BALB/c-nu mice	Used as traditional Chinese medicine component. Completed phase II of a clinical trial on pancreatic cancer
Shikonin^161,300^	↑	RIPK1 and RIPK3- dependent necroptosis	Various human cell lines	Used in traditional Chinese medicine as a wound healing ointment
Tanshinone IIA^193^	↑	Especially in the presence of caspase inhibitors forms RIPK1/RIPK3 complex	In human hepatocellular carcinoma HepG2 cells	Constituent of the traditional medicinal plant *Salvia miltiorrhiza*
Youdujing^164^	↑	Increases RIP1 expression	In ectocervical Ect1/E6E7 cell line	Traditional Chinese herbal formula
Wogonin^192^	↓	Inhibited RIPK1 by occupying the ATP-binding pocket	Inhibits necroptosis in cisplatin-induced AKI mouse model	Herbal compound, was found in *Scutellaria baicalensis*, ingredient of a Japanese herbal supplement

### Regulation the expression level of necrosome components

Drugs that control the promoters of RIPK3 or MLKL or modify the stability and degradation of these molecules can regulate necroptosis sensitivity. Interferons^27,29^, hypomethylating agents such as decitabine (5-aza-2′-deoxycytidine) and 5-azacytidine (used in Myelodysplastic syndromes and AML)^87^, histone deacetylase inhibitor valproic acid^158^ (VPA), anti-fungal miconazole^159^, traditional Chinese medicine drugs (shikonin^160,161^, resibufogenin^162^, bufalin^163^, youdujing^164^, emodin^165^), and components found in different plants (matrine^166^, genipine^167^, lycorine^168^, quercetin^169^, curcumol^170^, Bulnesia sarmientoi^171^) were all found to upregulate the expression of RIPK1 or RIPK3.

On the other hand, various inhibitors of the HSP90 have been documented to downregulate necroptosis (Kongensin A^172^, G-TPP^173^, geldanamycin^174^, gamitrinib^10^, DHQ3^175^ and 17-demethoxy-reblastatin^175^). Cyclosporine A^176^, Diacerein^177^ (Used in Europe and Asia to treat joint diseases), immunosuppressive and antiproliferative Rapamycin^178^ and traditional Chinese medicine such as patchouli alcohol^179^ have been also documented to reduce the expression of principal necroptotic mediators. Ex-527^180^ (which completed a phase II clinical trial in Huntington disease) regulates necroptosis through the inhibition of Sirt1 deacetylase.

### Regulation the activity of necrosome components

Beside the expression of necrosome components, the activity of these enzymes is also modified by various drugs. Promising specific inhibitors are currently being developed for the central molecules of necroptosis. RIPK1, RIPK3, and MLKL (reviewed in refs. ^181,182^) which may interfere with unwanted cell death and subsequent inflammation. Multiple second mitochondria-derived activator of caspase (SMAC) mimetics and TAK-1 (reviewed in ref. ^183,184^) inhibitors are being tested in clinical trials to activate necroptosis for therapeutical intervention, by restoring the sensitivity of apoptosis-resistant tumors to cell death. Since these drugs are reviewed elsewhere, we focus on currently available necroptosis regulators.

Drugs currently used for the treatment of different forms of tumors display anti-necroptotic activity (Dabrafenib^185,186^, Sorafenib^187,188^, Pazopanib^189^, Ponatinib^189^, and Carfilzomib^190^) as does the anti-epilepsy drug Phenhydan^191^. Phenytoin^51^ (a clinically used anti-convulsant) or herbal components such us wogonin^192^ and aucubin^193^ inhibit RIPK1 activity. All these drugs provide immediate translational potential to dampen necroptosis-driven tissue degradation. Presumably, these drugs will be additive to the above-mentioned necroptosis inhibitors which downregulate the expression of necrosome components.

On the other hand, radiation^194^, or chemotherapeutic agents such as anthracyclines and oxaliplatin^195^, cisplatin^196,197^, 5-fluorouracil^198^ or the pan-BCL-2 inhibitor Obatoclax^199^ (several phase two trials have been completed), traditional Chinese medicines such as resibufogenin^162^ (also tested in phase II of a clinical trial on pancreatic cancer), aucubin^193^, tanshinone^200^ or neoalbaconol^201^ have been documented to upregulate necroptosis. Based on current results, these drugs regulate the activity, and not the expression of necroptotic component. As a mono-therapy these group of necroptosis regulators could be ineffective in tumors that downregulate the level of RIPK3 or MLKL, but these medicines may increase the effect of the above listed mediators in combination therapy following the restoration of RIPK1 or RIPK3 expression in cancer cells.

### Regulation the signaling of necroptotic pathway

Some drugs regulate necroptosis by modulating the level or activity of partner molecules of the necrosome. For example, VPA induces the release of SMAC from mitochondria thereby upregulating necroptosis similarly to the widely tested SMAC mimetics. Dimethyl fumarate (DMF^202^, which is currently used in relapsing-remitting multiple sclerosis) induces necroptosis via downregulation of the negative regulators of necroptosis such as IAPs and cFLIPs. Aurora kinase inhibitors have been shown to directly induce necroptosis and stimulated intra-tumoral phosphorylation of MLKL^203^. Drugs antagonizing Trx1function as necroptosis inducers. PX-12^123^ (completed phase I of a clinical trial on advanced metastatic cancer) and DMF^204^ target TRX1 and have been shown to sensitize tumor cells to necroptosis.

Various drugs activate necroptosis via regulation of downstream components of necroptosis. Adiponectin receptor agonists^205^ (tested in various clinical trials), DMF^202^, neoablaconol^206^ induce ROS production. Lithium^126^ (clinically used for treating bipolar disorders) facilitates AKT-mTOR-mediated necroptosis, while dasatinib (used drug in CML) induces HMGB1-mediated necroptosis.

Necroptosis can be inactivated via the regulation of interacting partners of the necrosome or by downstream components, as well. The proteasome inhibitor Bortezomib^207^ (used in Multiple Myeloma treatment) and a HDAC inhibitor Vorinostat^208^ (approved for the treatment of Cutaneous T cell lymphoma) have been demonstrated to inhibit necroptosis through the upregulation of necroptosis inhibitors, sequentially stabilizing IAPs or increasing FLIP expression.

Various ROS scavengers have been implicated in the modulation of necroptosis^209,210^. Dexmedetomidine (used in moderate sedation) inhibits HMGB1 production^211^. Melatonin^212^ (used for jetlag sleep disorder) blocks PGAM5, while P110 is a selective inhibitor of Drp1^213^, therefore, these two drugs dampen the intensity of necroptosis via a well-documented PGAM-Drp1 pathway.

While there are no drugs on the market directly approved to regulate necroptosis, various medicines have the potential to both up and downregulate necroptosis, and to interact different levels of necroptosis signaling. Necroptosis has fundamental roles in various human diseases which makes it rational to try and apply the necroptosis regulator drugs in these syndromes.
